# Is There a Spatial Relationship between Urban Landscape Pattern and Habitat Quality? Implication for Landscape Planning of the Yellow River Basin

**DOI:** 10.3390/ijerph191911974

**Published:** 2022-09-22

**Authors:** Dike Zhang, Jianpeng Wang, Ying Wang, Lei Xu, Liang Zheng, Bowen Zhang, Yuzhe Bi, Hui Yang

**Affiliations:** 1 School of Foreign Languages, China University of Geosciences, Wuhan 430074, China; 2 Changjiang Institute of Survey, Planning, Design and Research, Wuhan 430014, China; 3 Key Laboratory of Changjiang Regulation and Protection of Ministry of Water Resources, Wuhan 430014, China; 4 Department of Land Resources Management, China University of Geosciences, Wuhan 430074, China; 5 Wuhan Economic and Technological Development Zone (Hannan District) Natural Resources and Planning Bureau, Wuhan 430056, China

**Keywords:** land-use simulation, landscape pattern, habitat quality, spatial autocorrelation, spatial regression, Yellow River Basin

## Abstract

The extent to which landscape spatial patterns can impact the dynamics and distribution of biodiversity is a key geography and ecology issue. However, few previous studies have quantitatively analyzed the spatial relationship between the landscape pattern and habitat quality from a simulation perspective. In this study, the landscape pattern in 2031 was simulated using a patch-generating simulation (PLUS) model for the Yellow River Basin. Then, the landscape pattern index and habitat quality from 2005 to 2031 were evaluated using the Fragstats 4.2 and the Integrated Valuation of Ecosystem Services and Tradeoffs (InVEST) model. Furthermore, we analyzed the spatial distribution characteristics and spatial spillover effects of habitat quality using spatial autocorrelation analysis. Finally, the spatial association between the landscape pattern index and habitat quality was quantitatively revealed based on a spatial lag model. The simulation results showed that: (1) from 2005 to 2031, the landscape of the Yellow River Basin would be dominated by grassland and unused land, and the areas of construction land and water body will increase significantly, while the area of grassland will decrease; (2) patch density (PD) and Shannon’s diversity index (SHDI) show significant increases, while edge density (ED), landscape shape index (LSI), mean patch area (AREA_MN), and contagion index (CONTAG) decrease; (3) from 2005 to 2031, habitat quality would decrease. The high-value areas of habitat quality are mainly distributed in the upper reaches of the Yellow River Basin, and the low-value areas are distributed in the lower reaches. Meanwhile, both habitat quality and its change rate present positive spatial autocorrelation; and (4) the spatial relationships of habitat quality with PD and COHESION are negative, while ED and LSI have positive impacts on habitat quality. Specifically, landscape fragmentation caused by high PD has a dominant negative influence on habitat quality. Therefore, this study can help decision makers manage future landscape patterns and develop ecological conservation policy in the Yellow River Basin.

## 1. Introduction

Land-use/cover change (LUCC) resulting from the interaction between human activities and the natural environment on temporal-spatial scales is directly expressed in the form of changes in surface landscape patterns [1]. Landscape patterns are defined as the spatial composition and configuration of land use. However, the rapid growth of industrialization and urbanization has intensified changes in land use and ecological issues, such as landscape fragmentation, occupation of natural habitat, environmental pollution, and loss of biodiversity [2], leading to a drastic reduction in habitat quality [3]. Habitat quality refers to the ability of the environment to provide conditions for human sustainable development and is an important support for species diversity and reproduction [4,5]. Numerous studies have shown that landscape pattern changes have a strong effect on habitat quality [6,7,8,9]. For example, landscape continuity, which is important for species to exchange materials, information, and energy flows, can lead to an increase in habitat quality. The increase in patches leads to landscape fragmentation, which is detrimental to animal migration and plant pollen dispersal and leads to a decrease in habitat quality. Most studies have utilized only the traditional linear equation approach to examine the influences of landscape pattern changes on regional habitat quality [10], ignoring the spatial autocorrelation and spatial spillover properties of habitat quality. However, spatial regression models are able to solve this problem, which can quantitatively analyze the spatial relationship between habitat quality and landscape pattern by considering spatial autocorrelation. The Yellow River Basin, an important ecological barrier in China’s ecological security strategy pattern, plays an important role in biodiversity conservation and healthy ecosystem maintenance. However, rapid socioeconomic development has led to the expansion of construction land and the loss of natural habitats in the Yellow River Basin. Therefore, it is necessary to simulate landscape patterns and analyze the spatiotemporal characteristics of habitat quality. More importantly, quantitative analysis of the spatial relationship between habitat quality and landscape patterns is of great importance for maintaining biodiversity and promoting sustainable development.

In recent years, many assessment methods have been applied to evaluate habitat quality, such Social Values for Ecosystem Services (SolVES), ARtificial Intelligence for Ecosystem Services (ARIES), Multi-scale Integrated Models of Ecosystem Services (MIMES), and Integrated Valuation of Ecosystem Services and Tradeoffs (InVEST) [11,12,13,14,15]. Among these models, the InVEST model is becoming a popular tool because it is more mature and easier to operate [16]. For example, Moreira et al. [17] adopted the InVEST model to assess the conservation status of Azorean natural habitats. Nematollahi et al. [18] evaluated the roads’ effects on the natural habitats of wild sheep based on the InVEST habitat quality module. Hack et al. [19] used the InVEST model to evaluate the impacts of built-up areas, roads, and water pollution on habitat quality. To conclude, the InVEST model can be applied to evaluate habitat quality in combination with habitat suitability and human activities and provides more detailed information about biodiversity [13,20]. Thus, the InVEST model is used in this study to evaluate the habitat quality of the Yellow River Basin.

Meanwhile, landscape patterns and biodiversity conservation have become one of the most popular issues in landscape ecology. The correlation of land use, landscape patterns, and ecosystems has drawn the attention of international scholars [21]. For example, Zhu et al. [22] used gray correlation analysis to explore the correlation between habitat quality and landscape pattern indexes in the eastern Qinghai-Tibet Plateau. Wu et al. [23] used Pearson’s correlation analysis methods to investigate the influential factors of habitat quality and showed that vegetation cover, intensity of human activity, and land-use change can cause a decline in habitat quality. Yushanjiang et al. [24] found that landscape pattern indexes were positively and negatively correlated with the ecosystem value in the Ebinur Lake Basin by using multiple linear regression models. However, most studies have ignored the spatial spillover effects of ecosystems, which are influenced not only by their own unit but also by the habitat quality of neighboring units. This will reduce the validity of conclusions. Noteworthy to mention is that most recent studies have begun to explore the spatial association between landscape patterns and ecosystems [1]. For instance, Zhu et al. [25] explored the effects of urbanization and landscape pattern changes on habitat quality in Hangzhou by using ordinary least squares (OLS) and geographically weighted regression (GWR) models. Chen et al. [26] used a multiscale spatial panel regression analysis approach to explore the impact of landscape patterns on ecosystem services. Thus, research on the mechanism of landscape pattern influence on habitat quality is gradually shifting from traditional linear correlation analysis or regressions to spatial econometric models. Quantitative analyses of the spatial association between landscape pattern and habitat quality can help to better understand the impact of changes in landscape pattern on habitat quality.

The abovementioned studies are very important guidelines for advancing habitat quality research, but they are all from the perspective of the past to analyze the spatiotemporal characteristics of habitat quality in the region. There is a growing need to explore the evolution of habitat quality from a simulation perspective, which can provide insights for ecological conservation planning and sustainable development. Cellular automata (CA) is the basis of many land-use simulation models. Researchers have proposed constrained CA, CA-Markov, the Conversion of Land Use and its Effects at Small regional extent (CLUE-S), and Future Land-Use Simulation (FLUS) models by improving the algorithms and techniques of CA and used these methods to predict habitat quality in the future. For example, Ding et al. [27] used the FLUS model to assess habitat quality changes in Dongying city in 2030 under multiple scenarios. Gomes et al. [28] simulated land use and habitat quality by using CA in Lithuania. Tang et al. [29] combined the CA-Markov and CLUE-S models to predict the evolution of habitat quality in Changli city. Li et al. [30] simulated urban growth and integrated habitat quality by using the SLEUTH model. However, most of the models were simulated based on each meta-cell scale and lacked the ability to simulate the evolution of patches with multiple land-use types. In this study, a patch-generating simulation (PLUS) model developed by Liang et al. [31] is adopted to simulate landscape pattern change. Compared with other CA-based models, PLUS has higher simulation accuracy and more realistic indicators of landscape patterns, which could provide more accurate quantitative assessment of the impact of landscape pattern on habitat quality.

The Yellow River Basin has been an important part of achieving balanced east-west and north-south development in China and plays an irreplaceable role in overall ecosystem health and biodiversity conservation in China. In the context of ecological protection and high-quality development, the evolution characteristics and spatial relationships of the landscape pattern and habitat quality in Yellow River Basin deserve unprecedented attention. Therefore, taking the Yellow River Basin as an example, this study quantitatively analyzes the spatial relationship between landscape pattern and habitat quality using a simulation approach to (1) simulate the future land use of Yellow River Basin based on the PLUS model and analyze the dynamic changes of the landscape pattern; (2) assess the spatiotemporal characteristics of habitat quality in Yellow River Basin using the InVEST model; (3) identify the spatial clusters of habitat quality and its rate of change from 2005 to 2031 based on univariate spatial autocorrelation; and (4) quantitatively evaluate the effect of the landscape pattern index on habitat quality based on the spatial lag model.

## 2. Materials and Methods

### 2.1. Study Area

**Figure 1:**
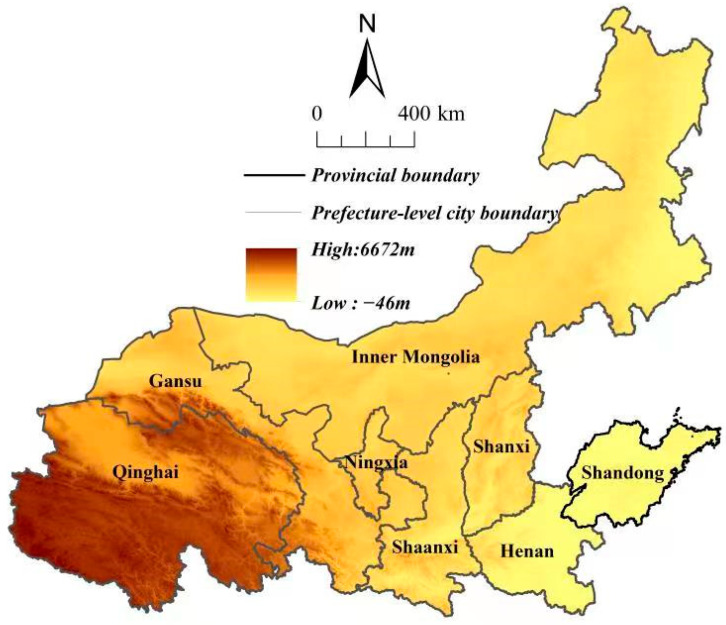
Location of the study area.

The Yellow River flows through nine provinces (i.e., Qinghai, Sichuan, Ningxia, Gansu, Shaanxi, Shanxi, Inner Mongolia, Henan, and Shandong). Eight of them are included in the boundary of the Yellow River Basin in this study (Figure 1), with Sichuan being excluded. Sichuan is often considered a part of the Yangtze River Economic Belt [32,33]. Generally, the Yellow River Basin has a high topography in the west and a low one in the east, with an average annual precipitation of 200–650 mm. The average altitude of the western headwaters region is above 4000 m, and the altitude of the central region is between 1000–2000 m. The Yellow River Basin is an important population catchment area and ecological security barrier in China. With abundant mineral and energy resources, the Yellow River Basin is one of the key areas of China’s socioeconomic development. It also serves as an ecological corridor that connects the Qinghai-Tibet Plateau, Loess Plateau, and North China Plain. Wetland resources are abundant, and species diversity is rich. There are also 12 national key ecological function areas in the region. Exploring the influences of landscape patterns on habitat quality is crucial for protecting biodiversity and improving the high-quality development level in Yellow River Basin.

### 2.2. Data Source

The basic data of this study include land-use maps, population density, Gross Domestic Product (GDP), nighttime lights, Digital Elevation Model (DEM), slope, aspect, distance from railway, distance from highway, distance from county center, distance from provincial government, precipitation, temperature, soil type, and Normalized Difference Vegetation Index (NDVI) (Table 1). The land-use data were obtained from the Chinese Academy of Sciences Data Center for Resources and Environmental Sciences (http://www.resdc.cn/, accessed on 12 May 2022), which were generated through remote sensing interpretation and manual visual interpretation from Landsat remote sensing images with an accuracy of 30 m. The landscape was classified into six land-use types, including cultivated land, forest, grassland, water body, construction land, and unused land. The DEM was obtained from the Geospatial Data Cloud platform (http://www.gscloud.cn/, accessed on 12 May 2022) and further used to derive the slope and aspect using the 3D Analyst tool in ArcGIS 10.2. Other data were obtained from the China Statistical Yearbook and the Resource and Chinese Academy of Sciences Data Center for Resources and Environmental Sciences, and all data were extracted as a 1000 × 1000 m raster dataset by using ArcGIS 10.2.

### 2.3. Methods

#### 2.3.1. Future LUCC Simulation Based on the PLUS Model

The PLUS model is a cutting-edge land-use simulation tool developed by Liang et al. in 2020 that includes two modules: the transformed rule mining framework (LEAS) based on a land expansion analysis strategy and the CA model (CARS) based on a multitype stochastic patch seeding mechanism [31]. It adopts the artificial neural network (ANN) algorithm to integrate natural and socioeconomic driving factors and simulate the suitability probability of each land-use type by combining the base period land-use data. Then, it uses the adaptive inertia competition mechanism based on roulette selection to solve the uncertainty and complexity of the interconversion of each type under the synergistic effects of natural and socioeconomic factors. The land-use demand, neighborhood factor, and conversion cost are set to simulate the land use at a future time point. The LUCC simulation based on the PLUS model involved five major steps.

1. Selection of driving factors for LUCC. The landscape pattern of the Yellow River Basin is affected not only by natural factors but also by socioeconomic and spatial location factors [34,35,36]. Considering the availability, diversity, and representativeness of the data, 14 driving factors were finally selected in this study, including rainfall, temperature, elevation, slope, aspect, population density, GDP density, nighttime light, soil type, NDVI, and distances to provincial governments, prefectural governments, railways, and highways.
2. Cost matrix and setting of restricted expansion areas. The cost matrix can be used to represent the cost of conversion between different land-use types (see Table S1 in Supplementary Materials). A value of 0 indicates that this land-use conversion is not allowed, while 1 means it is allowed [37]. In this study, the cost matrix and the restricted expansion area were set based on previous studies and realistic conditions. In reality, construction land is rarely converted to other land-use types. Therefore, this study assumes that the conversion of construction land to other land-use types is not allowed. To ensure food security, this study prohibits the conversion of cultivated land to unused land. To promote ecological protection, the 1000 m buffer zone along the main stream of the Yellow River was set as a restricted expansion area, and conversion of landscape types in this area was prohibited.
3. Setting of neighborhood weight parameters. The neighborhood weight parameter indicates the expansion intensity of each land-use type. The parameter ranges from 0 to 1, where values closer to 1 have stronger expansion abilities. In this study, the expansion intensity of each land-use type was determined based on the experience of existing studies and the characteristics of landscape evolution in the Yellow River Basin (see Table S2 in Supplementary Materials).
4. Land-use demand prediction. This study used Markov models to predict the land-use structure in 2031 based on the probability matrix of land-use changes from 2005–2018 and the current land-use development patterns in the Yellow River Basin.
5. Model validation. Based on the land-use data in 2005, we simulated the land use of the Yellow River Basin in 2018 using the model parameters specified above and compared it with the classified land-use map in 2018 from Landsat remote sensing images. The kappa coefficient and figure of merit (FoM) were used to verify the simulation accuracy. The validation results showed that the kappa coefficient and FOM were 0.84 and 0.28, respectively. The simulation accuracy achieved a high level, which indicated that the PLUS model is reliable for future land-use simulations in 2031.

#### 2.3.2. Landscape Pattern Indexes Analysis

The landscape pattern index is an important tool to analyze the spatiotemporal characteristics of landscape patterns by reflecting the composition and spatial configuration of the landscape structure and the evolution of landscape patterns. The study selects landscape pattern indexes based on the diversity, aggregation, and complexity of the landscape space. The selected landscape pattern indexes are patch density (PD), mean patch area (AREA_MN), edge density (ED), landscape shape index (LSI), Shannon’s diversity index (SHDI), patch cohesion index (COHESION), and contagion index (CONTAG) [38]. The specific calculation was performed by using Fragstats 4.2 software.

#### 2.3.3. Habitat Quality Evaluation

Stable habitat quality is a crucial basis for sustaining ecosystem biodiversity. In this study, the habitat quality module of the InVEST model was adopted to evaluate habitat quality [39,40]. As the researchers have established, higher levels of land use and socioeconomic activity pose greater threat to habitat conservation and are correlated with lower-quality habitat and vice versa [41]. The model combines the sensitivity of different land-use types to threat factors and with intensity of external threats. Specifically, cultivated land, construction land, and unused land are identified as the threat factors of habitat quality in this study. The model parameters, including the maximum stress distance, weight, type of spatial recession, and sensitivity of LUCC to habitat threat factors, are specified according to the model’s manual and expert experience (Table 2) [42,43].

The change rate in habitat quality is measured by the percentage change in regional habitat quality at the beginning and the end of a time period, expressed as follows:

(1) $$V=\left(P_{t1}-P_{t0}\right)/P_{t0}\times 100\%$$

In Equation (1), V is the change rate of habitat quality, with a negative value indicating decreasing habitat quality and vice versa; $P_{t0}$ and $P_{t1}$ are the initial and final values of habitat quality in the *t*-th year, respectively.

#### 2.3.4. Spatial Autocorrelation Analysis

A spatial autocorrelation approach was used to verify the spatial dependence and spatial spillover effects of habitat quality [44]. Univariate spatial autocorrelation analysis includes global and local autocorrelation analysis [45]. Moran’s I has been widely used for representing global autocorrelation, i.e., the overall clustering pattern. The value of Moran’s I ranges from −1 to 1. A higher Moran’s I value suggests a more significant positive spatial autocorrelation of habitat quality. To explore the local spatial association and type of spatial clusters of habitat quality in different prefecture-level cities, we used the local indicator of spatial association (LISA) [46]. In addition, the spatial clusters of habitat quality in this study were classified into four types, i.e., high-high (H-H), low-low (L-L), high-low (H-L), and low-high (L-H). Specifically, the H-H clusters mean that the prefecture-level city and its neighbors all had a high habitat quality that was higher than the average In the Yellow River Basin. The L-L clusters indicate that the prefecture-level city and its neighbors had a low habitat quality that was lower than the average. The H-L clusters indicate that the prefecture-level city and its neighbors had negative spatial autocorrelation of habitat quality. That is, the prefecture-level cities with high habitat quality were surrounded by the prefecture-level cities with low habitat quality and vice versa for the L-H clusters. Thus, the H-H clusters and L-L clusters indicated that the habitat quality of the area was similar to that of its neighbors. The formulas for global Moran’s I and local Moran’s I are shown as follows:

(2) $$I=\frac{\sum_{P=1}^{K}\sum_{q=1}^{k}W_{pq}\left(Y_{P}-\bar{Y}\right)\left(Y_{q}-\bar{Y}\right)}{S^{2}\sum_{p=1}^{k}\sum_{q=1}^{k}W_{pq}}$$

(3) $$I_{p}=\frac{Y_{p}-\bar{Y}}{S^{2}}\overset{k}{\underset{q=1}{\sum }}W_{pq}(Y_{p}-\bar{Y})$$

In Equations (2) and (3), I is the global Moran’s *I* for the whole area, and its value ranges from −1 to 1; $I_{p}$ is the local Moran’s I for prefecture-level city *p*; *Y_p_* and *Y_q_* are the habitat quality of prefecture-level cities *p* and *q*; $S^{2}$ is the discrete variance of *Y_q_*; $\bar{Y\;}$ is the average value of habitat quality; *k* is the number of prefecture-level cities; *W_pq_* is the spatial weight matrix, representing that prefecture-level city *p* is adjacent to prefecture-level city *q*, and the value of *W_pq_* is 1 if they are adjacent and otherwise 0.

#### 2.3.5. Spatial Regression Analysis

The spatial lag model (SLM) and spatial error model (SEM) are usually used for spatial regression analysis [47,48]. To determine whether SLM or SEM is more appropriate in this study, we used a Lagrange multiplier (LM) test and a robust Lagrange multiplier (RLM) test to verify by using OLS [45]. We found that both LM (lag) and its RLM (lag) are more significant than LM (error) and its RLM (error). Thus, we selected SLM to quantitatively analyze the influence of the landscape pattern indexes on habitat quality in this study. The OLS can be defined as follows:

(4) $$Q_{pt}=\beta L_{t}+\varepsilon$$

The SLM can be defined as follows:

(5) $$Q_{pt}=\rho \varpi_{1}Q_{pt}+\beta L_{t}+\varepsilon$$

The SEM can be defined as follows:

(6) $$Q_{pt}=\beta L_{t}+\gamma \varpi_{2}+\varepsilon$$

In Equations (4)–(6), $Q_{pt}$ is the habitat quality in prefecture-level city *p* in the *t*-th year; ρ, γ  is the spatial lag parameter and spatial error parameter; $\varpi_{1},\;\varpi_{2}$ is the spatial weight matrix of the lag terms and error terms, respectively; β is the parameter revealing the correlations between habitat quality and landscape pattern indexes; $L_{t}$ is the landscape pattern index in the *t*-th year; and ε is a constant.

## 3. Results

### 3.1. Spatiotemporal Characteristics of Landscape Patterns from 2005 to 2031

#### 3.1.1. Predicted Land-Use Changes

**Figure 2:**
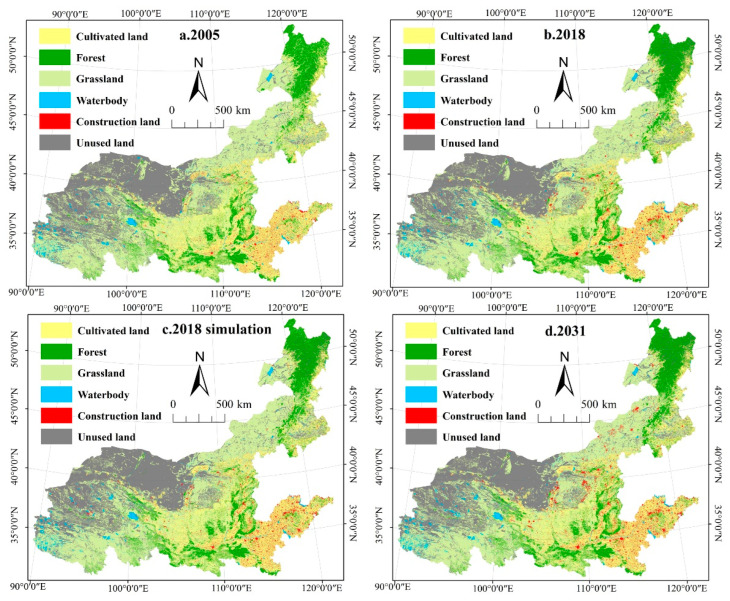
The land-use simulation map of the Yellow River Basin. (**a**). 2005 represents the land use of the Yellow River Basin in 2005; (**b**). 2018 represents the land use of the Yellow River Basin in 2018; (**c**). 2018 simulation represents the simulated land use of the Yellow River Basin in 2018; (**d**). 2031 represents the simulated land use of the Yellow River Basin in 2031.

The simulated land-use pattern of the Yellow River Basin in 2031 using the PLUS model is shown in Figure 2. Landscape change in the Yellow River Basin slows down from 2018 to 2031 compared to 2005–2018 (Table 3). The main landscape types in the Yellow River Basin were grassland and unused land, while construction land and water bodies accounted for the smallest proportion. From 2005 to 2031, landscape changes are mainly characterized by the transformation of cultivated land and grassland into construction land and forest. Specifically, from 2005 to 2018, the areas of cultivated land and grassland decreased, and the highest reduction by 3.10% was observed in grassland. Construction land, forest, water bodies, and unused land were all expanding, with construction land increasing by 37.48%. The predicted trend of landscape change from 2018 to 2031 is similar to that in 2005–2018, with the area of cultivated land and grassland showing a decreasing trend and the area of construction land, forest, water body, and unused land showing an increasing trend.

#### 3.1.2. Landscape Pattern Metrics

The landscape pattern indexes in the Yellow River Basin show different change trends from 2005 to 2031 (Table 4). In particular, the PD and SHDI increase continuously by 3.23% and 3.76%, respectively, from 2005 to 2031, indicating that the landscape in the Yellow River Basin would become more fragmented. In contrast, the AREA_MN and CONTAG decrease by 3.17% and 6.94%, respectively, which also demonstrates that the landscape would be more heterogenous. According to AREA_MN, the average size of patches within the landscape becomes smaller, indicating increasing fragmentation. The CONTAG index is used to describe the degree of clustering or extension trend of different patch types within the landscape. The reduction of CONTAG indicates that the number of patches with a certain dominant type of connectivity in the landscape is decreasing, and thus, the fragmentation of the landscape is growing. Meanwhile, the ED and LSI indexes slightly decrease by 0.55% and 0.48%, respectively, from 2005 to 2031. The decrease of ED and LSI indicates patches within the landscape are becoming more spatially aggregated. However, the ED and LSI increase from 2018 to 2031, reflecting the trend towards fragmentation and complexity of the landscape pattern change. In addition, the COHESION remains basically unchanged from 2005 to 2031.

From the perspective of landscape types (see Table S3 in Supplementary Materials), the LSI and PD of construction land, water bodies, unused land, and cultivated land show increasing trends, while the LSI and PD of grassland and forest generally decrease. There were no significant changes in the ED of cultivated land and forestland. The ED of water bodies, construction land, and unused land significantly increases, and the ED of grassland decreases. The AREA_MN and COHESION of water bodies, construction land, and forest increase significantly, and that of grassland and unused land does not change significantly.

### 3.2. Spatiotemporal Characteristics of Habitat Quality from 2005 to 2031

#### 3.2.1. Temporal Changes of Habitat Quality

Results indicate that the habitat quality in the Yellow River Basin displays a declining tendency from 2005 to 2031. The average habitat quality in the Yellow River Basin decreases by 0.98% and 1.05% from 2005 to 2018 and 2018 to 2031, respectively. From the perspective of landscape types (Table 5), the average habitat quality of forest and construction land remains stable, the average habitat quality of water body and grassland increased, and grassland has the largest increase, with a specific increase of 0.05%. The average habitat quality is highest in forests and grasslands, followed by water bodies and unused lands, and lowest in cultivated land and construction land, which is mainly because of the high habitat suitability of forests, grasslands, and water bodies, which are far from threat sources. Therefore, natural vegetation (i.e., forest, grassland, etc.) plays a vital role in maintaining the habitat quality of the Yellow River Basin.

#### 3.2.2. Spatial Evolution of Habitat Quality

**Figure 3:**
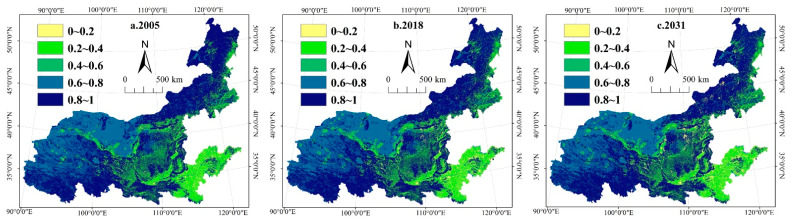
Distribution of habitat quality. (**a**). 2005 represents the habitat quality of the Yellow River Basin in 2005; (**b**). 2018 represents the habitat quality of the Yellow River Basin in 2018; (**c**). 2031 represents the habitat quality of the Yellow River Basin in 2031.

From 2005 to 2031, the spatial distribution pattern of habitat quality in the Yellow River Basin remains stable (Figure 3), and the overall habitat quality is high. In this study, habitat quality is classified as highest (0.8–1.0), high (0.6–0.8), medium (0.4–0.6), low (0.2–0.4), and lowest (0–0.2). Specifically, the highest-level and high-level habitat quality areas are mainly distributed in areas with high vegetation cover, such as the Qinghai-Tibet Plateau and northeastern Inner Mongolia. These areas are important ecological sources for maintaining regional ecological security, and urban expansion should be strictly limited. Medium-level habitat quality areas are concentrated in the Loess Plateau region represented by Ningxia, Gansu, and Shaanxi. Low-level and lowest-level habitat quality areas are mainly distributed in Henan, Shandong, and other provinces downstream of the Yellow River Basin and present a clustered pattern. These areas are the main distribution areas of cultivated land and towns and are also the areas with the highest intensity of human activities in the Yellow River Basin. The contradiction between socioeconomic development and ecological protection is prominent here, and urban development has already produced a strong duress on the surrounding habitats. Therefore, excessive growth of urban space in the future should be limited, especially excessive occupation of ecological land.

### 3.3. Spatial Clustering Characteristics and Spatial Relationships

#### 3.3.1. Univariate Spatial Autocorrelation Analysis

(1) Spatial autocorrelation of habitat quality

**Figure 4:**
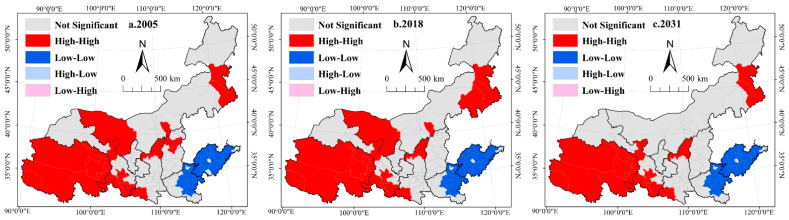
LISA cluster map of habitat quality in the Yellow River Basin. (**a**). 2005 represents the LISA cluster map of habitat quality of the Yellow River Basin in 2005; (**b**). 2018 represents the LISA cluster map of habitat quality of the Yellow River Basin in 2018; (**c**). 2031 represents the LISA cluster map of habitat quality of the Yellow River Basin in 2031.

To analyze the spatial distribution characteristics and spatial spillover effects of habitat quality in the Yellow River Basin, spatial autocorrelation analysis was performed for the habitat quality by using prefecture-level cities as units of analysis. The global Moran’s I values of each city in 2005, 2018, and 2031 are greater than 0.82 (0.8217, 0.8322, and 0.8248, respectively), and the *p*-values are less than or equal to 0.001, indicating that the spatial distribution of habitat quality in the Yellow River Basin exhibits significant positive spatial autocorrelations. In addition, according to the results of local spatial autocorrelation (Figure 4), habitat quality shows a clear bipolar clustering feature in space (i.e., high-high clusters and low-low clusters). The spatial clustering characteristics of habitat quality are similar in 2005, 2018, and 2031. The high-high clusters of habitat quality in the Yellow River Basin are concentrated in the Qinghai-Tibet Plateau, the southern Loess Plateau, and western and eastern Inner Mongolia. The low-low clusters of habitat quality are concentrated in Henan and Shandong in the lower reaches of the Yellow River Basin.

(2) Spatial autocorrelation of the rate of change in habitat quality

**Figure 5:**
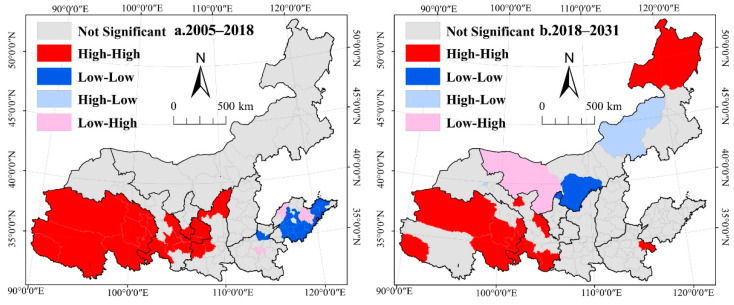
LISA cluster map of the rate of habitat quality change in the Yellow River Basin. (**a**). 2005–2018 represents the LISA cluster map of the rate of habitat quality change in the Yellow River Basin in 2005–2018; (**b**). 2018–2031 represents the LISA cluster map of the rate of habitat quality change in the Yellow River Basin in 2018–2031.

According to the global spatial autocorrelation analysis of the rate of change in habitat quality, the global Moran’s I values are 0.4280 and 0.3764 from 2005 to 2018 and 2018 to 2031, respectively. The results show that the spatial distribution of the rate of change in habitat quality in Yellow River Basin presents significant positive spatial autocorrelations from 2018 to 2031 (Figure 5). The H-H clusters of the rate of change in habitat quality in the Yellow River Basin are mainly concentrated in the plateau areas from 2005 to 2031. This result indicates that there is a significant improvement in habitat quality in the plateau region during this period. From 2005–2018, the H-H agglomeration areas of the rate of change in habitat quality are mainly distributed in most areas of Qinghai province and parts of Gansu and Shaanxi provinces. The L-L agglomeration areas of the rate of change in habitat quality are distributed in Shandong and parts of Henan, and there are no areas of H-L clusters. From 2018 to 2031, the H-H agglomeration areas of the rate of change in habitat quality are mainly distributed in most areas of Qinghai, the eastern part of Inner Mongolia, and parts of Gansu and Shaanxi provinces. The L-L agglomeration areas of the rate of change in habitat quality are distributed in Shandong and parts of Henan. The L-H agglomeration areas of the rate of change in habitat quality are distributed in the western part of Inner Mongolia. The H-L agglomeration areas of the rate of change in habitat quality are distributed in the eastern part of Inner Mongolia. From 2005 to 2018, the spatial aggregation characteristics of high or low values of the rate of change in habitat quality in the Yellow River Basin are closely related to human activities, land-use policies, and ecosystem protection engineering projects. From 2018 to 2031, the spatial aggregation characteristics of habitat quality change rates are also influenced by the specification of cost matrix and restricted areas in the land-use simulation. The results can provide data support for biodiversity conservation and ecological priority area setting in the Yellow River Basin.

#### 3.3.2. Spatial Regression Analysis

The above analysis confirmed the spatial autocorrelation of habitat quality. Therefore, we can further explore the spatial spillover effect of habitat quality and its influential factors by using a spatial regression model. In this study, the habitat quality of 95 prefecture-level cities in the Yellow River Basin was included in the model as the dependent variable, while the independent variables in the model were the landscape pattern indexes. In addition, multicollinearity diagnosis of landscape pattern indexes was used to eliminate the presence of multicollinearity in multiple landscape pattern indexes by using IBM SPSS Statistics. Four factors with VIF < 8 were selected as independent variables of the model: PD, ED, LSI, and COHESION.

As can be seen, the log likelihood of the spatial lag model is larger than that of the OLS model (AIC and SC values are smaller than those of the OLS model) (see Table S4 in Supplementary Materials), which indicates that the fitting degree of the spatial lag model is better than that of the OLS model. Almost all the independent variables in the spatial lag model (Table 6) are significant (*p* < 0.05) from 2005 to 2031. The spatial lag regression results demonstrate that the spatial relationships between habitat quality and PD as well as COHESION are negative, while ED and LSI had a positive impact on habitat quality. Meanwhile, a 1% increase in PD led to decreases of 4.622%, 3.926%, and 4.041% in habitat quality in 2005, 2018, and 2031, respectively. The impact of ED is positive, but its effect size decays over time, as a 1% increase in ED can lead to increases of 0.031%, 0.036%, and 0.002% in habitat quality in 2005, 2018, and 2031, respectively. The impact of LSI fluctuates over time and increases substantially from 2018 to 2031, e.g., a 1% increase in LSI can lead to increases of 0.005%, 0.003%, and 0.038% in habitat quality in 2005, 2018, and 2031, respectively. In addition, the impact of COHESION on habitat quality is negative and decreases over time. The results show that PD is the dominant driving factor of the decrease in habitat quality with the largest magnitude of effect. As displayed in Table 6, the PD increases during the study period, demonstrating that the landscape in the Yellow River Basin would become more fragmented. Specifically, the PD of water bodies and cultivated land increases. Therefore, landscape fragmentation due to higher PD has a strong influence on ecosystem structure, ecological processes, and biodiversity and causes degradation of habitat quality.

## 4. Discussion

### 4.1. Spatiotemporal Characteristics of Habitat Quality and Landscape Pattern

In this study, we examined spatiotemporal evolution characteristics of landscape pattern and its impact on regional habitat quality and then combined the PLUS and InVEST models to predict future habitat quality levels in the Yellow River Basin. The evaluation of current habitat quality and projection of future habitat quality in the Yellow River Basin are of great significance for ecological protection and high-quality development in the Yellow River Basin.

In general, the landscape of the Yellow River Basin was dominated by grassland and unused land. The area of construction land in the east was significantly larger than that in the west. From 2005 to 2018, the area of arable land and grassland decreased, while the area of construction land, forest, water body, and unused land increased. Meanwhile, PD and SHDI increased significantly in the Yellow River Basin, while ED, LSI, AREA_MN, and CONTAG decreased, and COHESION remained basically unchanged. The predicted trend of landscape pattern changes from 2018 to 2031 is basically the same as it was from 2005 to 2018. Although the proportion of built-up land area in the total landscape area of the Yellow River Basin is low, the expansion of built-up land in recent decades has caused the destruction of forest, grassland, water, and other habitat landscapes. This phenomenon is more obvious in the population and economic agglomeration areas in the lower reaches of the Yellow River Basin (e.g., Henan and Shandong, etc.). This is because areas with more intensified human activities and massive land-use changes have rapid population and economic growth and great demands for housing, transportation, and public facilities, leading to the occupation of many natural resources such as grasslands, water bodies, and forests and increasing the degree of landscape diversity and fragmentation.

In addition, we assessed habitat quality in the Yellow River Basin. It was found that the habitat quality of the western and northern Yellow River Basin along the Qinghai-Tibet–Inner Mongolia was relatively high. The main reason is that the region has good natural endowment and less construction occupation. It has also gradually established a nature reserve management system with national parks as the main body, nature reserves as the basis, and various nature parks as supplements. More importantly, due to the intervention of afforestation, water conservation, and other ecological protection measures, the number of forests and water bodies with high habitat quality in this area continues to increase. However, habitat quality was at a low level in the middle and lower reaches of the Yellow River Basin. The population and economic agglomeration effect is more obvious in this region. The urban expansion continues to occupy natural resources, leading to the degradation of habitat quality. The influence of the continuous expansion of construction land on the degradation of habitat quality is mainly the reduction of cultivated land, forest, and other landscape land area [49]. At the same time, severe landscape fragmentation reduces landscape connectivity and affects the overall regional habitat, which is particularly critical to the quality of regional habitat.

### 4.2. Impact of Landscape Pattern Change on Habitat Quality

The results showed that the influence of landscape pattern change on regional habitat quality could not be ignored, and its impact direction and magnitude vary largely in different regions. Therefore, it is of great significance to analyze the effect of landscape pattern on habitat quality for regional landscape planning and ecological sustainable development. Spatial regression was used to quantitatively analyze the correlation between landscape pattern index and habitat quality in the Yellow River Basin. The results showed that the change of landscape pattern had an important effect on habitat quality in the Yellow River Basin. Landscape pattern indices (PD, ED, LSI, and cohesion) had significant effects on habitat quality. The regression coefficients for LSI and ED were both positive, indicating that increased LSI and ED improved habitat quality, while the regression coefficients for PD and cohesion were negative, indicating that increased PD and cohesion resulted in decreased habitat quality. Despite the positive contribution of LSI and ED to habitat quality, both LSI and ED values fluctuated during the study period. There was the greatest negative impact of PD on habitat quality, and the PD value increased over time, which was a major factor in the decline of habitat quality in the Yellow River Basin. In general, the effect of PD on habitat quality reduction was greater than that of LSI and ED enhancement, and the increase of PD implied that the landscape was more fragmented, and the landscape connectivity was weakened, which was related to the decrease of biodiversity and habitat quality. Some relevant studies support our findings. For example, Hu et al. used Geographically and Temporally Weighted Regression (GTWR) and Multiscale Geographic Weighted Regression (MGWR) methods to analyze the driving mechanisms of landscape patterns on habitat quality and found that an increase in landscape connectivity in the urban center of Nanjing significantly improved habitat quality, while an increase in fragmentation in high habitat areas reduced habitat quality [50].

As part of spatial planning and land-use construction in the Yellow River Basin, it is necessary to coordinate the relationship between development and protection to improve regional habitat quality. It is important for the government to maintain the landscape integrity of natural habitats (such as forests, rivers, and wetlands) as much as possible, arrange agricultural landscapes reasonably, and improve the landscape diversity of urban construction areas [25]. Specific measures can be adopted, including delineating ecological protection red lines and delimiting permanent primary farmland, managing high- and low-quality areas of ecological space [51], and establishing pocket parks and green corridors.

### 4.3. Strengths and Limitations

In this study, the PLUS model was used to simulate the LUCC of the Yellow River Basin in 2031, with 2018 as the base period. Numerous studies adopting the CA-based model have focused mainly on improving technical modeling procedures rather than simulating the detailed patches of multiple land-use types that evolve over time. The PLUS model developed by Liang et al. [31] has a powerful ability to simulate the evolution of land-use types at patch scale. It has been confirmed that the PLUS model has higher simulation accuracy and landscape pattern indicators that were closer to the real landscape than the other CA-based models. This is essential for accurate quantitative assessment of the impact of future landscape patterns on habitat quality and thus the development of policies to manage future land use and landscape patterns in the Yellow River.

At the same time, spatial autocorrelation models and spatial regression models are used to analyze the spatiotemporal characteristics of habitat quality and its response to landscape pattern changes. Various ecological processes often lead to nonrandom spatial distributions of land use, landscape, and biodiversity and show some dependence on spatial patterns. Thus, spatial autocorrelation analysis is crucial for understanding how ecological variables are related and vary in time and space, which can then be used to understand and predict ecological processes and functions. In addition to traditional factors, spatial autocorrelation is also an important factor that influences habitat quality and landscape pattern, but this factor is often overlooked. In previous studies, linear models are often used to analyze the relationship between landscape pattern and habitat quality, which cannot capture the spatial dependence and spillover effects due to spatial autocorrelation. Spatial regression models and spatial autocorrelation models are used to overcome this shortcoming in this study.

However, there are several limitations in this study. First, the InVEST model was used to evaluate the habitat quality of the Yellow River Basin by accumulating the effects of threat factors. Despite this, InVEST does not take into account the interaction between the threat factors, as their cumulative impact on habitat quality is not the same as their simple accumulation [29]. Second, this study only analyzed the impact of seven landscape pattern indexes that were recognized as significant, while other related landscape pattern indexes were not comprehensively considered.

## 5. Conclusions

This study analyzes the spatiotemporal characteristics of landscape patterns and habitat quality, explores the spatial association between the landscape pattern indexes and habitat quality, and proposes reasonable suggestions to protect and improve habitat quality from the perspective of landscape pattern protection.

Firstly, the results showed that the landscape of the Yellow River Basin is dominated by grassland and unused land, and the area of construction land in the east is significantly greater than that in the west. From 2005 to 2031, the areas of cultivated land and grassland decreased, while the areas of construction land, forest, water bodies, and unused land increased. Then, it was found that a significant increase in PD and SHDI will occur in the Yellow River Basin, while ED, LSI, AREA_MN, and CONTAG will decrease, and COHESION remains almost unchanged. In general, landscape heterogeneity increases, and landscape connectivity decreases. In addition, the habitat quality in the Yellow River Basin shows a continuous decrease trend during the study period, but the change is not drastic. This is because the landscape pattern evolution has both enhanced and diminished effects on habitat quality, which offset each other to a certain extent. Forests, grasslands, and water bodies have the highest habitat quality among landscape types, while construction lands have the lowest. Finally, a spatial lag regression model was further applied to quantitatively assessed the effects of the landscape pattern on habitat quality. The results show that PD and COHESION have significant negative impacts on habitat quality, whereas ED and LSI have significant positive impacts. Landscape fragmentation due to high PD exerts the most significant negative effect on habitat quality. Therefore, we should consider enhancing the connectivity of habitats in landscape planning and limiting the fragmentation of ecological land caused by the uncontrolled expansion of construction land in order to achieve biodiversity conservation and ecological sustainability.

## Figures and Tables

**Table 1:** Data information and sources.

Data Type	Data Name	Data Source and Preprocessing
Land-use data	Basic land-use data at 30 m (2005)	Chinese Academy of Sciences Data Center for Resources and Environmental Sciences (http://www.resdc.cn, accessed on 12 May 2022)
	Basic land-use data at 30 m (2018)	
Driving factors Of LUCC	Spatial distribution of population density	
	Spatial distribution of GDP	
	Nighttime light	
	Rainfall	
	Temperature	
	Soil type	
	NDVI	
	DEM	Geospatial Data Cloud (http://www.gscloud.cn/, accessed on 12 May 2022)
	Slope	Extract from DEM by Using ArcGIS 10.2
	Aspect	
	Distance to railway	Extract by using ArcGIS Euclidean distance function
	Distance to highway	
	Distance to provincial governments	
	Distance to prefectural governments	

**Table 2:** Input data used for InVEST model.

Threat Factors	Maximum Duress Distance (km)	Weights	Land-Use Types					
			Cultivated Land	Forest	Grassland	Water Body	Construction Land	Unused Land
			**Habitat suitability**					
			0.3	1	1	0.7	0.3	0.6
			**Threat factors**					
Cultivated land	4	0.6	0	0.6	0.8	0.5	0	0.6
Construction land	8	0.4	0.8	0.4	0.6	0.4	0	0.4
Unused land	6	0.5	0.4	0.2	0.6	0.2	0.1	0

**Table 3:** Landscape type structure of the Yellow River Basin from 2005 to 2031 (unit: 10^4^ km^2^).

Time	Cultivated Land	Forest	Grassland	Water Body	Construction Land	Unused Land
2005	54.46	36.17	120.79	5.84	6.43	75.50
2018	53.88	37.29	117.05	6.42	8.84	75.71
2031	53.75	38.17	113.89	6.94	10.64	75.79
2005–2018	−0.58	1.12	−3.74	0.58	2.41	0.21
2005–2018	−1.07%	3.10%	−3.10%	9.93%	37.48%	0.28%
2018–2031	−0.13	0.88	−3.16	0.53	1.80	0.08
2018–2031	−0.24%	2.36%	−2.70%	8.26%	20.36%	0.11%

**Table 4:** Landscape pattern indexes of Yellow River Basin.

Landscape Pattern Indexes	2005	2018	2031	2005–2018	2018–2031	2005–2031
PD	0.0526	0.0535	0.0543	1.71%	1.50%	3.23%
ED	5.8669	5.7729	5.8346	−1.60%	1.07%	−0.55%
LSI	257.6114	253.6939	256.3796	−1.52%	1.06%	−0.48%
AREA_MN	1902.8027	1869.4666	1842.4869	−1.75%	−1.44%	−3.17%
CONTAG	34.7186	33.3331	32.3093	−3.99%	−3.07%	−6.94%
COHESION	99.6430	99.6361	99.6225	−0.01%	−0.01%	−0.02%
SHDI	1.4385	1.4696	1.4926	2.16%	1.57%	3.76%

**Table 5:** Average habitat quality of landscape types in the Yellow River Basin from 2005–2031.

Landscape Types	2005	2018	2031	Average
Cultivated land	0.2998	0.2997	0.2997	0.2997
Forest	0.9960	0.9960	0.9960	0.9960
Grassland	0.9881	0.9887	0.9886	0.9885
Water body	0.6943	0.6944	0.6947	0.6945
Construction land	0.0000	0.0000	0.0000	0.0000
Unused land	0.5997	0.5996	0.5996	0.5996
Yellow River Basin	0.7388	0.7315	0.7253	0.7319

**Table 6:** Regression results of SLM.

Variable	2005		2018		2031	
	Habitat Quality	*p*	Habitat Quality	*p*	Habitat Quality	*p*
PD	−4.6219 ***	0.0000	−3.9258 ***	0.0000	−4.0412 ***	0.0000
ED	0.0313 ***	0.0002	0.0362 **	0.0018	0.0024 *	0.0471
LSI	0.0046 ***	0.0000	0.0026 *	0.0335	0.0381 **	0.0014
COHESION	−0.0381 ***	0.0000	−0.0184 *	0.0233	−0.0170 *	0.0366
CONSTANT	4.1951 ***	0.0000	2.1218 **	0.0100	1.9812 *	0.0170
Spatial lag term	0.2734 ***	0.0000	0.4368 ***	0.0000	0.4349 ***	0.0000
Measures of fit						
Log likelihood	91.1599		63.5760		61.8309	
AIC	−170.3200		−115.1520		−111.6620	
SC	−154.9970		−99.8288		−96.3386	
R2	0.7814		0.6449		0.6328	

Note: *** *p* ≤ 0.001, ** *p* ≤ 0.01, and * *p* ≤ 0.05. AIC, Akaike information criterion; SC, Schwartz’s.

## Data Availability

The remote sensing data were obtained from the Chinese Academy of Sciences Data Center for Resources and Environmental Sciences (http://www.resdc.cn, accessed on 12 May 2022).

## Supplementary Materials

The following supporting information can be downloaded at: https://www.mdpi.com/article/10.3390/ijerph191911974/s1, Table S1: Conversion cost matrix; Table S2: Neighborhood weight parameters for different land use types; Table S3: Landscape pattern indexes of landscape types in Yellow River Basin; Table S4: Regression results of the ordinary least-squares (OLS) method.
