# Supplementary material for: Is There a Spatial Relationship between Urban Landscape Pattern and Habitat Quality? Implication for Landscape Planning of the Yellow River Basin
Source: Int J Environ Res Public Health. 2022 Sep 22;19(19):11974. doi: 10.3390/ijerph191911974 (PMC9565473; doi:10.3390/ijerph191911974)
Supplement: Supplementary file 1 [file ijerph-19-11974-s001.zip › ijerph-1894266-Supplementary.pdf]

Table S1. Conversion cost matrix.

	**a**	**b**	**c**	**d**	**e**	**f**
a	1	1	1	1	1	0
b	1	1	1	1	1	1
c	1	1	1	1	1	1
d	1	1	1	1	1	1
e	0	0	0	0	1	0
f	1	1	1	1	1	1

Note: a-f, respectively represent cultivated land, forest, grassland, water body, construction land and unused land.

Table S2. Neighborhood weight parameters for different land use types.

Land use types	Water body	Cultivated land	Forest	Grassland	Construction land	Unused land
Weight of neighborhood	0.7	0.5	0.5	0.8	1	0.4

Table S3. Landscape pattern indexes of landscape types in Yellow River Basin.

Landscape types	year	PD	ED	LSI	AREA_MN	COHESION
Waterbody	2005	0.0056	0.4088	127.6674	350.9270	73.5657
	2018	0.0060	0.4442	132.8501	359.6457	74.3394
	2031	0.0063	0.4784	137.5674	369.6991	76.0415
Unused Land	2005	0.0074	1.9267	167.1328	3397.2656	99.7471
	2018	0.0083	1.9790	171.6106	3059.9830	99.7183
	2031	0.0082	1.9815	171.7325	3072.8157	99.7179
Grassland	2005	0.0115	4.4329	303.8013	3526.0640	99.7790
	2018	0.0106	4.0275	280.4727	3703.0054	99.8085
	2031	0.0107	4.0135	283.2525	3545.1441	99.7999
Forest	2005	0.0095	1.7138	215.0723	1271.1590	98.7878
	2018	0.0090	1.7075	211.1227	1378.7600	98.8539
	2031	0.0090	1.7031	208.2346	1416.0144	98.8647
Cultivated Land	2005	0.0080	2.5948	265.1179	2262.1890	99.5193
	2018	0.0083	2.593	266.0368	2173.6717	99.3995
	2031	0.0083	2.586	265.7014	2173.1048	99.4007
Construction land	2005	0.0106	0.6569	194.7382	203.4787	51.9910
	2018	0.0114	0.7947	200.8655	259.9494	67.8466
	2031	0.0117	0.9066	208.7688	303.0348	73.7986

Table **S4**. Regression results of the ordinary least-squares (OLS) method.

Variable	2005		2018		2031	
	**Habitat quality**	**P**	**Habitat quality**	**P**	**Habitat quality**	**P**
PD	-5.9440***	0.0000	-6.3096***	0.0000	3.6251***	0.0004
ED	0.0407***	0.0000	0.0598***	0.0000	-6.4938***	0.0000
LSI	0.0058***	0.0000	0.0044**	0.0037	0.0041**	0.0057
COHESION	-0.0490***	0.0000	-0.0338***	0.0008	0.0631***	0.0000
CONSTANT	5.4240***	0.0000	3.8513**	0.0002	-0.0316**	0.0015
Moran's I(error)	0.2899***	0.0000	0.2459***	0.0001	0.2349***	0.0003
Lagrange Multiplier(lag)	16.7871***	0.0000	25.7752***	0.0000	24.3044***	0.0000
Robust LM(lag)	5.9603*	0.0146	14.5499***	0.0001	14.0467***	0.0002
Lagrange Multiplier(error)	15.6195**	0.0001	11.2371***	0.0008	10.2581**	0.0014
Robust LM(error)	4.7927*	0.0286	0.0118	0.9135	0.0004	0.9838
Measures of fit						
Log likelihood	82.6520		49.5745		48.5718	
AIC	-155.3040		-89.1490		-87.1436	
SC	-142.5350		-76.3796		-74.3742	
R^2	0.7219		0.4989		0.4901	

Note: *** p ≤ 0.001, ** p ≤ 0.01, and * p ≤ 0.05. AIC—Akaike information criterion, and SC—Schwartz's criterion.
